# Ciliate Genome Sequence Reveals Unique Features of a Model Eukaryote

**DOI:** 10.1371/journal.pbio.0040304

**Published:** 2006-08-29

**Authors:** Richard Robinson

One reason to sequence the genomes of non-human organisms is to better understand our similarities and differences. And, at first sight, it is hard to imagine a eukaryote more different from humans than *Tetrahymena thermophila*. A relative of *Paramecium*, this single-celled creature has a strong but flexible exterior covered with rows of cilia; but it is inside where things seem to get really alien. Each cell contains not one but two nuclei: a micronucleus, which contains only five chromosomes, and a macronucleus, which has more than 200.

Biologists have long known that the micronucleus contains the DNA reserved for reproduction, and that the macronucleus arises from the micronucleus and controls the cell’s other functions. During macronucleus formation (which happens each time the cells mate), each of the five chromosomes splinters into multiple fragments, which in turn replicate to form many copies of the resulting smaller chromosomes. In a new study, Jonathan Eisen and a team of over 50 scientists report the full sequence of the macronuclear genome.

The authors began by isolating DNA from purified macronuclei (no mean feat in itself), and performed a “shotgun” sequence, splitting the DNA into millions of fragments, sequencing each of these, and then reconstructing the whole by using computers to match overlaps. They estimate that they have captured more than 95% of the genome, and conclude it is 105 million base pairs in length. The exact number of chromosomes is still at issue, though the authors present evidence that it lies between 185 and 287, and, based on the number of telomeres, is probably about 225.

**Figure:**
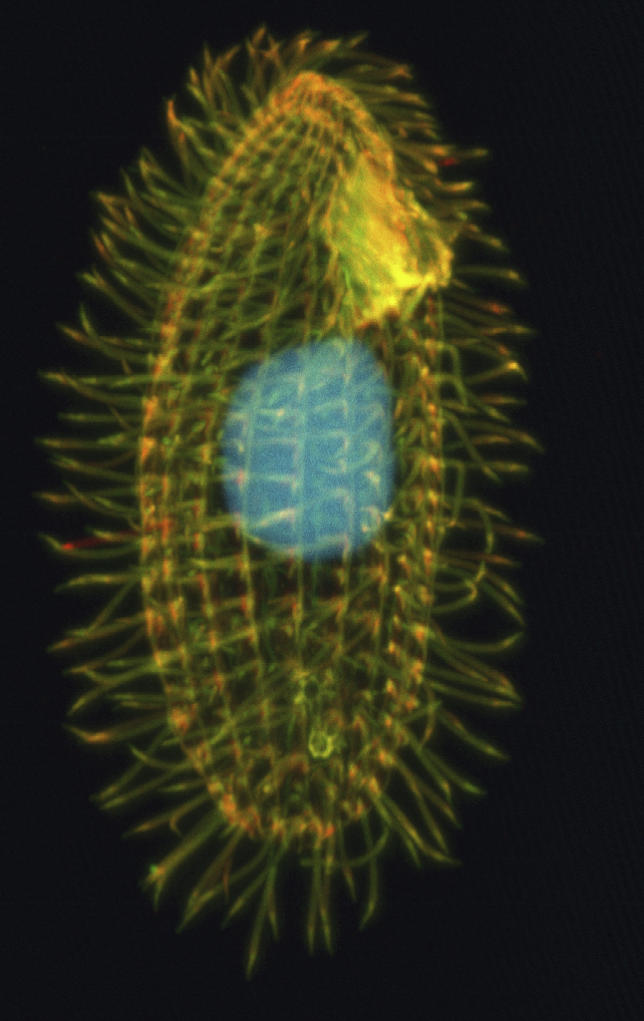
The genome sequence of the single-celled ciliate *Tetrahymena thermophila* sheds light on early eukaryotic evolution.

*T. thermophila* macronuclear chromosomes, unlike those in the micronucleus and other species, are highly unusual because they appear to lack centromeres, the regions that link chromosomal replicants and then guide their separation during mitosis and meiosis. This makes some sense, since the macronucleus undergoes neither process. Furthermore, they contain much less repetitive DNA than most other eukaryotes—about 2% of the total DNA, versus over 50% in humans—partly because most repetitive DNA is jettisoned during the formation of the macronucleus, when about 15% of micronuclear genomic DNA is excised. The authors provide evidence that excision targets not only repeated elements per se but also foreign DNA (such as “selfish” mobile DNA transposons) in particular, indicating the importance of this process in maintaining the integrity of the expressed genome from such outside invasions.

Sequencing the genome also allowed the authors to address a nagging evolutionary question, namely the timing of plastid acquisition in the alveolates, a group of three related phyla: the ciliates (including *Tetrahymena*), the apicomplexans (parasites that cause malaria, among other diseases), and the dinoflagellates (ocean-dwelling photosynthetic protozoans). Plastids, such as the chloroplast, are organelles descended from what were once free-living cyanobacteria; typically, many of the genes of such an endosymbiont are shifted into the host nucleus, as they have been in the apicomplexans and dinoflagellates. *T. thermophila* has no plastids, but it has been suggested that its ancestors did. The authors discovered no remnants of plastid genes within *T. thermophila*, strongly suggesting that plastid acquisition occurred after the other two groups split off from the ciliates.

All told, the genome contains over 27,000 protein-coding genes, more than naively expected for a single-celled species and comparable to the number in humans. Certain gene families appear to have expanded significantly in *T. thermophila*, indicating the likely importance of the processes carried out by the proteins each family encodes. An example is the presence of over 300 genes for voltage-gated ion channels, which control membrane transport, a key function of this free-living, single-celled creature. Previous analysis of gene structure showed that *T. thermophila* uses only one stop codon (UGA) during protein synthesis, compared to the three that are standard in most eukaryotes; the unused ones instead encode glutamine. As in many other organisms, UGA itself is also used in some genes to encode the amino acid selenocysteine, making T. thermophila the only known organism to translate all 64 codons.

The authors also wish to sequence the micronucleus genome, which should provide insights into *T. thermophila* biology that is unavailable from the macronucleus alone. A key component of the project is that all of the data have been made publicly available without restrictions throughout the project, allowing the scientific community to freely analyze the genome of this organism even prior to this publication.

